# Global, regional, and national burdens of myocarditis, 1990–2019: systematic analysis from GBD 2019: GBD for myocarditis

**DOI:** 10.1186/s12889-023-15539-5

**Published:** 2023-04-19

**Authors:** Yue-Wen-Ying Wang, Run-Ben Liu, Cheng-Yang Huang, Hao-Yang Li, Zhi-Xin Zhang, Xiao-Zheng Li, Jia-Ling Liu, Chao Zhang, Xing Xiong, Yu-Ming Niu

**Affiliations:** 1 Center for Evidence-Based Medicine and Clinical Research, Taihe Hospital, Hubei University of Medicine, No.32, Renmin South Road, Shiyan, 442000 China; 2 Department of Emergency, Taihe Hospital, Hubei University of Medicine, No.32, South Renmin Road, Shiyan, 442000 China; 3 Department of Stomatology, Center for Evidence-Based Medicine and Clinical Research, Gongli Hospital of Shanghai Pudong New Area, Shanghai, 200135 China

**Keywords:** Myocarditis, Global Burden of Disease, Disability-adjusted life years, Social-Demographic Index, Estimated annual percentage change

## Abstract

**Objectives:**

Myocarditis, a health-threatening heart disease, is attracting increasing attention. This systematic study was conducted to study the prevalence of disease through the trends of incidence, mortality, disability-adjusted life years (DALYs) over the last 30 years, which would be helpful for the policymakers to better the choices for reasonable decisions.

**Methods:**

The global, regional, and national burdens of myocarditis from 1990–2019 were analyzed by using the 2019 Global Burden of Disease (GBD) database. This study on myocarditis produced new findings according to age, sex, and Social-Demographic Index (SDI) by investigating DALYs, age-standardized incidence rate (ASIR), age-standardized death rate (ASDR), and corresponding estimated annual percentage change (EAPC).

**Results:**

The number of myocarditis incidence increased by 62.19%, from 780,410 cases in 1990 to 1,265,770 cases in 2019. The ASIR decreased by 4.42% (95%CI, from -0.26% to -0.21%) over the past 30 years. The number of deaths from myocarditis increased by 65.40% from 19,618 in 1990 to 324,490 in 2019, but the ASDR was relatively stable over the investigated period. ASDR increased in low-middle SDI regions (EAPC=0.48; 95%CI, 0.24 to 0.72) and decreased in low SDI regions (EAPC=-0.97; 95%CI, from -1.05 to -0.89). The age-standardized DALY rate decreased by 1.19% (95%CI, from -1.33% to -1.04%) per year.

**Conclusions:**

Globally, the ASIR and DALY for myocarditis decreased and the ASDR was stable over the past 30 years. The risk of incidences and death cases increased with age. Measures should be taken to control the risk of myocarditis in high-burden regions. Medical supplies should be improved in the high-middle SDI regions and middle SDI regions to reduce the deaths from myocarditis in these regions.

## Introduction

Myocarditis has been a major public health issue for a long time and is the predominant cause of sudden cardiac death, especially among young people [1]. Globally, it has been proclaimed that there will be 10.2–105.6 people with myocarditis per 100,000 populations. It is also estimated that there are 1.8 million incidences of myocarditis each year [1, 2]. The most recent study shows the incidence in 2017 was 3,071,000, with a 59.6% increase from 1990 and the death cases increased from 27,120 in 1990 to 46,490 in 2017 [2]. In 2017, there were 131,376(95%UI 90,113–183,001) years lived with disability (YLDs) [2]. From 1990 to 2017, myocarditis remained essential world-wide public health problems and the increasing numbers of the prevalent cases, deaths, YLDs and years of life lost (YLLs) call for effective preventive prevention and treatment programs [2].

Myocarditis, also known as inflammatory cardiomyopathy, is characterized by myocardial infiltration with inflammatory cells and non-ischemic myocytic necrosis, which is a type of heart disease caused by regional or diffuse inflammation, leading to the global burden of the disease mainly by sudden death and dilated cardiomyopathy [3–5]. The outcomes from myocarditis are usually favorable; however, complications can be observed in some cases and include dilated cardiomyopathy [6], cardiogenic shock, and sudden cardiac death [5]. When the occurrence of the cardiac failure or arrhythmia comes, acute myocarditis should be taken attention to [7]. In Sharifrazi’s study, approximately 20% of under-40-year-old adults suffer from accidental death after developing myocarditis [4]. Moreover, the different causes of myocarditis and the heterogeneity of clinical symptoms pose significant challenges to the identification and diagnosis of this disease. Furthermore, the procession of the disease is multistage and multicompartmental. Among all the patients in UK hospital, 4% of all which developed acute myocarditis [8].

The Global Burden of Disease (GBD) study uses measurements such as incidence, deaths, YLL, and disability-adjusted life year (DALY), serving as a platform for researchers and health workers around the world to obtain a comprehensive understanding of a particular disease [9]. GBD 2019 included 369 diseases and injuries and 87 risk factors and combinations of risk factors for 204 countries and territories, with the aim of providing strategies for different areas and identifying causes of threatening diseases all over the world [10, 11]. To date, there are only a few recent GBD studies that comprehensively discuss the situation with myocarditis. Consequently, more detailed studies and analyses are necessary, which may help policymakers allocate and optimize the limited medical resources to reduce the risk of this disease.

## Methods

### Overview

GBD 2019 contains many indexes such as DALYs, providing open and free information for health workers, which plays a key role in the comprehension of epidemiological trends [12]. The study involved a combined and unremitting effort worldwide and will update annually, classified by age, sex, and Social-Demographic Index (SDI). We extracted the information on myocarditis from the study and analyzed the DALYs, ASIR, and ASDR from 21 regions [13, 14].

### Data and manipulation

Information on myocarditis from 1990-2019 was extracted from the Global Health Data Exchange (GHDx) query tool (http://ghdx.healthdata.org/gbd-results-tool). R software (version 4.1.1, packages involving readxl, ggsci, cowplot, showtext, patchwork, ggplot2, gridExtra, plyr, ggpubr, viridis, expression, maps, RColorBrewer and reshape2) was employed for all statistical analyses.

### Statistical method

DALYs for a disease or health condition are the sum of the YLLs to due to premature mortality and the years lived with disability (YLD) due to prevalent cases of the disease or health condition in a population [14–16].

Age-standardized rates were used in the present study to eliminate interruptions of age composition, implement a process of initial data, and attempt to show the information and discoveries in a manner close to that of the real situation. Firstly, calculating the value of a certain age group multiplies its corresponding rate of that group. Then, by summing population (${a}_{i}$) or the weight (${w}_{i}$) of the subgroup, the ASRs were obtained, i.e., $ASR=\frac{{\sum }_{i=1}^{A}{a}_{i}{w}_{i}}{{\sum }_{i=1}^{A}{a}_{i}}\times 100000$. EAPC is used to estimate the trends of the ASRs, which follows this formula, i.e., $y=\alpha +\beta x+\varepsilon$, where y is equal to $\mathrm{ln}ASR$, and x is equal to calendar year. Calculation formula for EAPC is EAPC = 100 × (exp(β) − 1) and through the regression model, its 95% confidence interval (CI) can be attained. EAPC = 100 × (exp(β) − 1) and its 95% confidence interval (CI) can be obtained. The correlation measure of relationship between the EAPC and ASIR/ASDR/DALY/SDI were employed using the Pearson method. There is a very strong association if the coefficient ranged from 0.8 to 1, a strong (or moderate) from 0.5 to 0.8, fair (or weak) from 0.2 to 0.5, and poor (or very weak) when less than 0.2. The *P* value <0.05 was statistically significant. Overall consideration was paid to the value of EAPC and the upper and lower level of 95% confidence interval (CI) demarcation, and based on all these factors, the changing trend of ASR can be identified. If the value of EAPC and the lower level of 95%CI were both greater than zero, the ASR was considered to be increasing, but if the value of EAPC and the upper level of 95%CI were both less than zero, the ASR was considered to be decreasing. Except for the two situations mentioned above, the ASR was considered to be stable with the passage of time [17, 18].

## Results

### Global estimates of myocarditis in 1990 and 2019

Across 204 countries and territories, there were 780,410 (95%UI, 620,640–951,210) incidences of myocarditis and 19,618 (95%UI, 15,686–26,772) deaths in 1990. The On the contrary, in 2019, there were 1265,770 (95%UI, 1021,670–1531,510) incidences and 32,449 (95%UI, 23,164–37,087) deaths (Tables 1 and 2). In addition, the ASIR decreased globally from 1990 to 2019 (16.74 per 100,000 persons in 1990 and 16.00 per 100,000 persons in 2019) (Table 1) and the ASDR decreased form 0.46 per 100,000 persons in 1990 to 0.43 per 100,000 persons in 2019 (Table 2). Furthermore, there were an estimated 981,400 (95%UI, 706,950–1578,960) DALYs and with an age-standardized DALY rate of 18.29 per 100,000 persons (95%UI, 13.81–27.58) in 1990, whereas the DALYs in 2019 were 977,240 (95%UI, 803,760–1126,800) with an age-standardized DALY rate of 12.81 per 100,000 persons (95%UI, 10.53–14.72) (Table 3).

**Table 1:** Incident cases and ASIR of myocarditis in 1990 and 2019, and temporal trends

	**1990**		**2019**		**1990-2019** **EAPC No.(95%CI)**
	Incident cases No.*10^**3**^**(95% UI)**	**ASIR per 100,000 No.(95% UI)**	Incident cases No.*10^**3**^**(95% UI)**	**ASIR per 100,000 No.(95% UI)**	
**Overall**	780.41 (620.64-951.21)	16.74 (13.46-20.34)	1265.77 (1021.67-1531.51)	16 (13.01-19.28)	-0.23 (from -0.26 to -0.21)
**Sex**					
**Female**	324.88 (258.72-397.17)	13.66 (11.04-16.67)	537.86 (435.58-651.42)	13.1 (10.62-15.84)	-0.23 (from -0.27 to -0.2)
**Male**	455.54 (363.9-557.22)	20.06 (16.21-24.31)	727.91 (585.21-879.25)	19.06 (15.51-22.89)	-0.25 (from -0.27 to -0.22)
**Socio-demographic index**					
**High SDI**	172.59 (138.52-210.56)	18.72 (15.07-22.8)	245.4 (200.09-298.32)	17.75 (14.71-21.22)	-0.41 (from -0.49 to -0.34)
**High-middle SDI**	182.86 (145.26-222.76)	16.58 (13.32-20.18)	269.56 (215.85-328.11)	16.01 (12.94-19.34)	-0.16 (from -0.18 to -0.14)
**Middle SDI**	229.79 (182.04-283.57)	16.39 (13.25-19.96)	379.02 (302.6-462.08)	15.79 (12.79-19.1)	-0.16 (from -0.17 to -0.14)
**Low-middle SDI**	137.07 (109.45-168.1)	15.93 (12.9-19.29)	245.19 (195.46-298.49)	15.68 (12.73-18.97)	-0.064 (from -0.069 to -0.058)
**Low SDI**	57.73 (45.98-71.2)	15.35 (12.43-18.68)	125.95 (100.11-155.46)	15.29 (12.38-18.59)	-0.0154 (from -0.0162 to -0.0146)
**Region**					
**Andean Latin America**	4.03 (3.24-4.93)	14.05 (11.38-17.11)	8.42 (6.8-10.22)	14.07 (11.41-17.1)	0.011 (0.005-0.018)
**Australasia**	3.6 (2.86-4.38)	16.51 (13.25-19.98)	6.22 (5-7.53)	16.45 (13.31-19.83)	-0.02 (from -0.04 to -0.01)
**Caribbean**	4.33 (3.48-5.24)	14.21 (11.5-17.19)	7.03 (5.69-8.56)	14.19 (11.48-17.16)	-0.0037 (from -0.0043 to -0.0031)
**Central Asia**	8.77 (7.01-10.69)	14.84 (11.86-17.95)	12.5 (9.93-15.27)	14.87 (11.88-17.98)	0.01 (0.008-0.011)
**Central Europe**	21.13 (16.81-25.81)	16.3 (13.13-19.83)	24.6 (19.63-30.18)	16.48 (13.27-20.05)	-0.004 (-0.037-0.029)
**Central Latin America**	18.38 (14.77-22.57)	15.06 (12.18-18.32)	36.44 (29.39-44.45)	14.97 (12.11-18.2)	-0.02 (from -0.022 to -0.019)
**Central Sub-Saharan Africa**	5.62 (4.43-6.93)	14.59 (11.72-17.82)	13.52 (10.6-16.74)	14.52 (11.67-17.7)	-0.017 (from -0.021 to -0.013)
**East Asia**	192 (151.52-238.07)	17.92 (14.47-21.97)	283.46 (228.98-346.68)	16.86 (13.73-20.36)	-0.28 (from -0.32 to -0.23)
**Eastern Europe**	42.7 (34.02-51.95)	17.64 (14.19-21.42)	46.06 (36.71-56.34)	17.74 (14.28-21.53)	0.016 (0.014-0.018)
**Eastern Sub-Saharan Africa**	20.02 (15.81-24.71)	15.26 (12.33-18.61)	44.33 (35-54.8)	15.25 (12.32-18.59)	-0.007 (from -0.009 to -0.005)
**High-income Asia Pacific**	37.57 (30.16-45.81)	20.66 (16.78-24.99)	54.82 (43.35-67.64)	20.07 (16.38-24.09)	-0.2 (from -0.24 to -0.17)
**High-income North America**	63.62 (50.75-77.84)	19.91 (16.01-24.29)	87.79 (73.29-103.53)	18.21 (15.37-21.45)	-0.83 (from -1 to -0.66)
**North Africa and Middle East**	31.37 (24.64-39.06)	12.01 (9.68-14.61)	64.13 (50.74-79.13)	12.05 (9.72-14.66)	0.018 (0.016-0.019)
**Oceania**	0.76 (0.6-0.94)	15.3 (12.32-18.49)	1.61 (1.29-1.99)	15.3 (12.31-18.5)	0.0014 (-0.0001-0.0029)
**South Asia**	133.98 (106.04-165.19)	16.28 (13.2-19.69)	259.99 (206.92-317.66)	16.18 (13.12-19.58)	-0.0225 (from -0.0232 to -0.0217)
**Southeast Asia**	57.11 (45.43-70.28)	15.14 (12.27-18.41)	96.38 (76.49-117.56)	15.14 (12.26-18.38)	-0.004 (from -0.006 to -0.002)
**Southern Latin America**	7.25 (5.84-8.78)	15.34 (12.43-18.64)	11.57 (9.37-14.07)	15.36 (12.45-18.66)	0.007 (-0.003-0.018)
**Southern Sub-Saharan Africa**	6.33 (5.07-7.77)	15.72 (12.72-19.06)	10.73 (8.52-13.1)	15.73 (12.72-19.08)	0.004 (0.001-0.007)
**Tropical Latin America**	19.02 (15.18-23.25)	15.76 (12.72-19.19)	36.76 (29.67-44.86)	15.72 (12.68-19.15)	-0.0084 (from -0.009 to -0.0078)
**Western Europe**	81.39 (65.53-99.3)	17.56 (14.19-21.33)	109.54 (87.4-134.98)	17.47 (14.21-21.14)	-0.06 (from -0.09 to -0.03)
**Western Sub-Saharan Africa**	21.43 (17.05-26.31)	15.48 (12.54-18.87)	49.89 (39.56-61.47)	15.37 (12.45-18.71)	-0.026 (from -0.031 to -0.021)

*ASIR* Age-standardized incidence rate, *EAPC* Estimated annual percentage change, *CI* Confidence interval, *SDI* Sociodemographic indices, *UI* Uncertainty interval

**Table 2:** Deaths and ASDR of myocarditis in 1990 and 2019, and temporal trends

	**1990**		**2019**		**1990-2019** **EAPC No.(95% CI)**
	Death cases No.*10^**2**^**(95% UI)**	**ASDR per 100,000 No.(95% UI)**	Death cases No.*10^**2**^**(95% UI)**	**ASDR per 100,000 No.(95% UI)**	
**Overall**	196.18 (156.86-267.72)	0.46 (0.38-0.6)	324.49 (231.64-370.87)	0.43 (0.31-0.5)	-0.09 (-0.38-0.2)
**Sex**					
**Female**	94.41 (63.98-142.53)	0.42 (0.30-0.59)	159.2 (104.95-190.93)	0.38 (0.25-0.45)	-0.26 (-0.58-0.06)
**Male**	101.77 (80.22-143.75)	0.5 (0.41-0.67)	165.29 (112.09-192.38)	0.48 (0.32-0.57)	0.07 (-0.18-0.33)
**Socio-demographic index**					
**High SDI**	23.21 (18.65-29.31)	0.26 (0.21-0.33)	42.56 (31.31-49.18)	0.27 (0.21-0.31)	0.12 (-0.12-0.36)
**High-middle SDI**	55.04 (43.63-71.5)	0.59 (0.45-0.74)	98.16 (70.64-114.74)	0.56 (0.4-0.65)	-0.06 (-0.62-0.49)
**Middle SDI**	77.95 (61.6-118.96)	0.67 (0.54-0.94)	116.42 (73.77-139.47)	0.59 (0.35-0.72)	-0.07 (-0.3-0.15)
**Low-middle SDI**	28.49 (16.88-39.34)	0.39 (0.28-0.47)	50.56 (32.14-60.17)	0.41 (0.24-0.5)	0.48 (0.24-0.72)
**Low SDI**	11.39 (5.67-19.14)	0.31 (0.21-0.44)	16.63 (11.81-24.62)	0.24 (0.15-0.37)	-0.97 (from -1.05 to -0.89)
**Region**					
**Andean Latin America**	0.63 (0.33-0.87)	0.27 (0.15-0.37)	0.66 (0.48-0.91)	0.12 (0.09-0.16)	-3.09 (from -3.28 to -2.89)
**Australasia**	0.74 (0.62-1.01)	0.36 (0.3-0.48)	1.18 (0.95-1.63)	0.3 (0.25-0.42)	-0.91 (from -1.38 to -0.43)
**Caribbean**	1.21 (0.69-2.44)	0.38 (0.24-0.66)	1.73 (1.19-2.52)	0.36 (0.24-0.56)	-0.13 (from -0.21 to -0.05)
**Central Asia**	2.5 (2.09-3.46)	0.47 (0.38-0.64)	6.2 (4.79-9.52)	0.78 (0.61-1.11)	2.55 (1.79-3.32)
**Central Europe**	12.39 (7.95-15.84)	1.05 (0.69-1.34)	22.47 (15.91-27.56)	1.12 (0.8-1.36)	-0.69 (from -1.12 to -0.26)
**Central Latin America**	1.39 (1.06-1.66)	0.12 (0.09-0.13)	3.17 (2.19-3.97)	0.14 (0.09-0.17)	0.43 (0.31-0.56)
**Central Sub-Saharan Africa**	1.51 (0.57-3.6)	0.35 (0.21-0.53)	1.89 (0.9-3.73)	0.24 (0.08-0.5)	-1.33 (from -1.39 to -1.28)
**East Asia**	89.04 (71.04-134.45)	1.07 (0.81-1.68)	135.04 (79.08-167.35)	0.9 (0.53-1.11)	-0.16 (-0.49-0.18)
**Eastern Europe**	8.54 (5.46-10.58)	0.41 (0.24-0.53)	15.64 (8.87-20.47)	0.53 (0.33-0.67)	0.7 (0.57-0.82)
**Eastern Sub-Saharan Africa**	5.1 (2.05-9.61)	0.24 (0.11-0.37)	4.73 (1.92-8.99)	0.14 (0.05-0.33)	-1.89 (from -2.01 to -1.76)
**High-income Asia Pacific**	5.26 (3.78-6.04)	0.33 (0.22-0.38)	7.81 (4.37-10.26)	0.21 (0.16-0.25)	-1.76 (from -1.85 to -1.66)
**High-income North America**	4.8 (3.96-7.3)	0.17 (0.14-0.25)	11.11 (7.33-12.98)	0.26 (0.18-0.3)	1.76 (1.2-2.33)
**North Africa and Middle East**	16 (9.38-33.08)	0.52 (0.34-0.85)	17.18 (12.33-30.55)	0.36 (0.26-0.65)	-1.19 (from -1.24 to -1.15)
**Oceania**	0.17 (0.08-0.3)	0.3 (0.17-0.44)	0.41 (0.19-0.67)	0.33 (0.18-0.5)	0.56 (0.47-0.64)
**South Asia**	13.82 (9.73-18.18)	0.24 (0.16-0.33)	28.33 (20.36-38.02)	0.21 (0.15-0.29)	-0.41 (from -0.48 to -0.34)
**Southeast Asia**	12.17 (8.37-18.81)	0.42 (0.28-0.53)	18.15 (12.62-23.63)	0.36 (0.23-0.44)	-0.56 (from -0.74 to -0.37)
**Southern Latin America**	1.22 (0.85-1.54)	0.28 (0.19-0.35)	1.93 (1.49-2.48)	0.24 (0.19-0.32)	-0.64 (from -0.75 to -0.52)
**Southern Sub-Saharan Africa**	1.18 (0.72-1.74)	0.3 (0.2-0.38)	1.38 (1.03-2.07)	0.22 (0.16-0.33)	-1.45 (from -1.65 to -1.25)
**Tropical Latin America**	3.1 (2.36-4.44)	0.28 (0.23-0.41)	6.66 (5.38-10.05)	0.3 (0.25-0.46)	0.14 (-0.22-0.49)
**Western Europe**	12.02 (8.52-15.39)	0.25 (0.18-0.32)	33.34 (16.81-44.97)	0.34 (0.19-0.43)	1.63 (0.7-2.57)
**Western Sub-Saharan Africa**	3.38 (1.48-5.49)	0.41 (0.17-0.70)	5.49 (3.83-7.43)	0.24 (0.17-0.31)	-2.32 (from -2.57 to -2.07)

*ASIR* Age-standardized incidence rate, *EAPC* Estimated annual percentage change, *CI* Confidence interval, *SDI* Sociodemographic indices, *UI* Uncertainty interval

**Table 3:** DALY and age-standardized DALY rate for myocarditis in 1990 and 2019, and temporal trends

	**1990**		**2019**		**1990-2019** **EAPC No.(95%CI)**
	DALY No.*10^**3**^**(95% UI)**	**Age-standardized DALY Rate per 100,000 No.(95% UI)**	DALY No.*10^**3**^**(95% UI)**	**Age-standardized DALY Rate per 100,000 No.(95% UI)**	
**Overall**	981.4 (706.95-1578.96)	18.29 (13.81-27.58)	977.24 (803.76-1126.8)	12.81 (10.53-14.72)	-1.19 (from -1.33 to -1.04)
**Sex**					
**Female**	443.11 (268.42-798.45)	16.39 (10.40-27.86)	406.89 (314.88-485.71)	10.39 (8.15-12.51)	-1.54 (from -1.71 to -1.37)
**Male**	538.29 (397.27-845.37)	20.14 (15.47-29.74)	570.34 (430.96-693.8)	15.21 (11.44-18.35)	-0.91 (from -1.04 to -0.79)
**Socio-demographic index**					
**High SDI**	95.26 (83.74-122.95)	11.97 (10.52-15.53)	124.68 (97.58-140)	11.63 (9.24-12.97)	-0.05 (-0.31-0.22)
**High-middle SDI**	225.19 (188.89-322.48)	21.17 (17.74-29.91)	236.16 (188.9-283.7)	15.39 (12.24-18.54)	-1.12 (from -1.35 to -0.88)
**Middle SDI**	425.94 (304.21-742.73)	25.58 (19.32-41.28)	346.19 (269.34-425.31)	15.81 (12.12-19.05)	-1.52 (from -1.66 to -1.39)
**Low-middle SDI**	160.73 (78.17-268.04)	14.05 (8.24-19.74)	178.99 (131.7-210.6)	11.51 (8.27-13.44)	-0.6 (from -0.76 to -0.43)
**Low SDI**	73.76 (26.97-149.82)	12.08 (6.69-18.73)	90.6 (64.78-128.07)	8.78 (6.37-12.81)	-1.1 (from -1.18 to -1.02)
**Region**					
**Andean Latin America**	2.73 (1.2-4.23)	7.98 (4.06-11.17)	2.12 (1.6-2.84)	3.49 (2.62-4.65)	-3.16 (from -3.35 to -2.97)
**Australasia**	3.59 (3.06-4.77)	18.11 (15.31-24.14)	4.21 (3.49-5.75)	13.34 (11.1-18.06)	-1.29 (from -1.69 to -0.89)
**Caribbean**	7.04 (3.08-18.18)	18.76 (9.05-45.08)	7.63 (4.36-14.27)	17.09 (9.31-33.57)	-0.16 (from -0.27 to -0.06)
**Central Asia**	10.95 (9.1-14.89)	17.71 (14.63-24.7)	24.27 (18.26-38.65)	26.7 (20.54-41.35)	2.05 (1.32-2.79)
**Central Europe**	33.03 (22.73-39.99)	27.56 (18.86-33.05)	42.76 (28.63-53.76)	25.68 (17.61-31.76)	-0.83 (from -1.09 to -0.58)
**Central Latin America**	8.36 (6.4-10.51)	4.89 (3.79-5.92)	12.5 (9.45-16.14)	5.25 (3.92-6.77)	0.21 (0.08-0.33)
**Central Sub-Saharan Africa**	10.09 (2.56-28.83)	15.22 (6.86-31.79)	10.33 (5.6-19.98)	9.2 (4.57-17.6)	-1.85 (from -1.91 to -1.79)
**East Asia**	466.82 (344.9-763.53)	41.66 (31.81-66.61)	351.71 (224.73-415.23)	25.17 (16.67-29.43)	-1.57 (from -1.85 to -1.28)
**Eastern Europe**	25.14 (20.34-37.47)	11.3 (9.31-16.17)	40.52 (30.05-50.89)	16.52 (12.98-22.05)	0.93 (0.77-1.1)
**Eastern Sub-Saharan Africa**	39.01 (11.94-81.31)	14.14 (6.39-24.17)	32.11 (14.41-56.2)	7.47 (3.34-14.01)	-2.19 (from -2.31 to -2.07)
**High-income Asia Pacific**	23.4 (20.04-30.54)	14.81 (12.35-18.98)	19.43 (15.31-22.68)	9.54 (8.26-12.05)	-1.76 (from -1.86 to -1.65)
**High-income North America**	29.43 (24.43-41.26)	11.02 (9.18-15.42)	49.34 (35.7-56.72)	14.6 (10.72-16.65)	1.28 (0.8-1.77)
**North Africa and Middle East**	109.81 (57.8-260.31)	25.72 (15.08-54.1)	87.02 (59.87-154.96)	15.28 (10.67-27.02)	-1.64 (from -1.7 to -1.57)
**Oceania**	1.19 (0.5-2.22)	15.75 (7.73-26.27)	2.69 (1.16-4.73)	17.74 (8.38-29.45)	0.6 (0.5-0.69)
**South Asia**	66.36 (40.97-95.2)	7.16 (5.05-9.45)	108.37 (85.25-140.12)	6.64 (5.18-8.62)	-0.27 (from -0.32 to -0.23)
**Southeast Asia**	63.24 (37.63-130.48)	14.57 (9.79-25)	63.63 (48.81-95.96)	10.58 (8.19-15.14)	-1.1 (from -1.28 to -0.92)
**Southern Latin America**	5.54 (4.25-7.29)	11.36 (8.69-14.82)	5.46 (4.48-7.4)	7.91 (6.47-10.93)	-1.47 (from -1.55 to -1.39)
**Southern Sub-Saharan Africa**	6.55 (3.57-11.4)	12.81 (7.84-19.51)	6.9 (5.11-9.89)	9.18 (6.84-13.08)	-1.26 (from -1.37 to -1.14)
**Tropical Latin America**	18.7 (13.16-26.37)	12.35 (9.06-17.42)	22.56 (18.43-33.3)	11.02 (8.83-16.26)	-0.4 (from -0.64 to -0.15)
**Western Europe**	34.03 (26.27-44.05)	8.4 (6.42-11.05)	54.67 (33.99-66.47)	8.74 (5.71-10.29)	0.31 (-0.16-0.78)
**Western Sub-Saharan Africa**	16.39 (6.62-28.41)	10.69 (5.12-16.89)	29 (19.47-41.64)	7.59 (5.48-9.94)	-1.44 (from -1.62 to -1.25)

*ASIR* Age-standardized incidence rate, *EAPC* Estimated annual percentage change, *CI* Confidence interval, *SDI* Sociodemographic indices, *UI* Uncertainty interval

### Global trends in myocarditis from 1990 to 2019

**Fig. 1:**
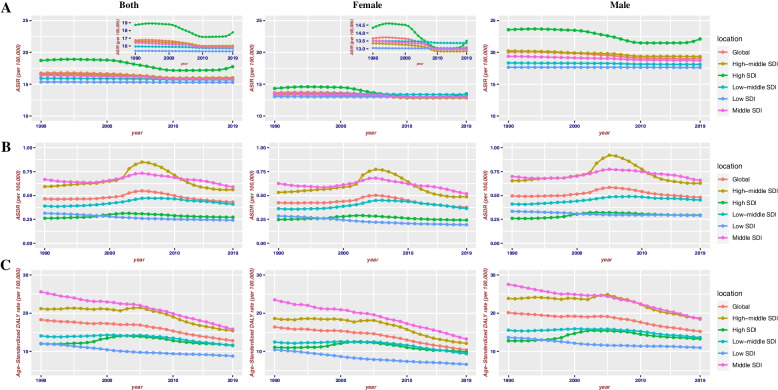
Change trends of ASIR, ASDR, and DALY rate of myocarditis among different SDI quintiles

**Fig. 2:**
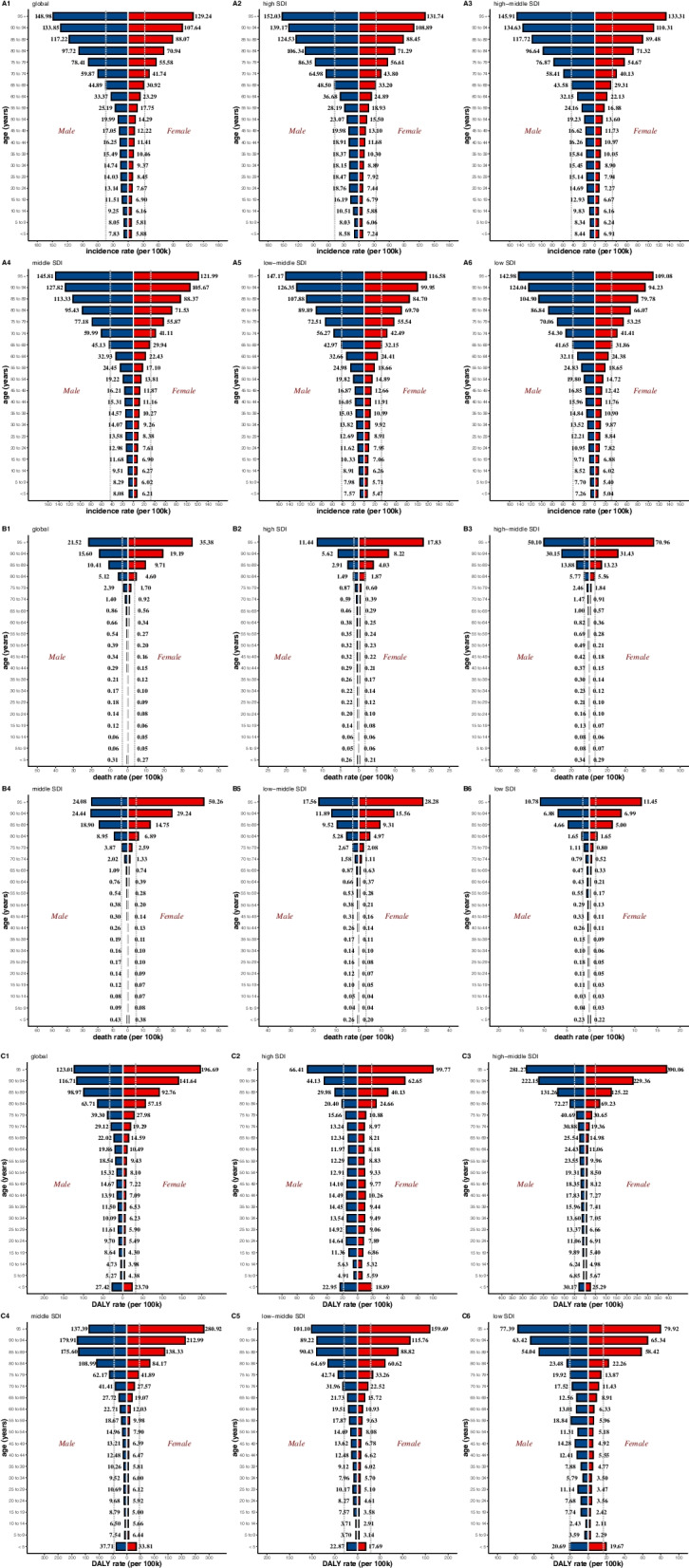
Incidence rate, death rate and DALY rate of myocarditis with respect to sex and different SDI regions in 2019. Note: A1, global incidence rate; A2, incidence rate in high SDI regions; A3, incidence rate in high-middle SDI regions; A4, incidence rate in middle SDI regions; A5, incidence rate in low-middle SDI regions; A6, incidence rate in low SDI regions; B1, global death rate; B2, death rate in high SDI regions; B3, death rate in high-middle SDI regions; B4, death rate in middle SDI regions; B5, death rate in low-middle SDI regions; B6, death rate in low SDI regions; C1, global DALY rate; C2, DALY rate in high SDI regions; C3, DALY rate in high-middle SDI regions; C4, DALY rate in middle SDI regions; C5, DALY rate in low-middle SDI regions; C6, DALY rate in low SDI regions

**Fig. 3:**
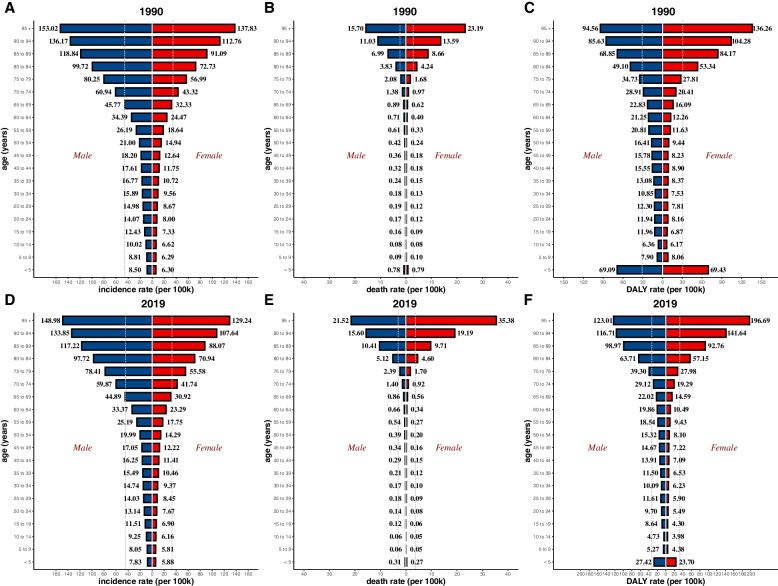
Global incidence rate, death rate and DALY rate of myocarditis with respect to sex and age in 1990 and 2019. Note: **A**, global incidence rate in 1990; **B**, global death rate in 1990; **C**, global DALY rate in 1990; **D**, global incidence rate in 2019; **E**, global death rate in 2019; **F**, global DALY rate in 2019

**Fig. 4:**
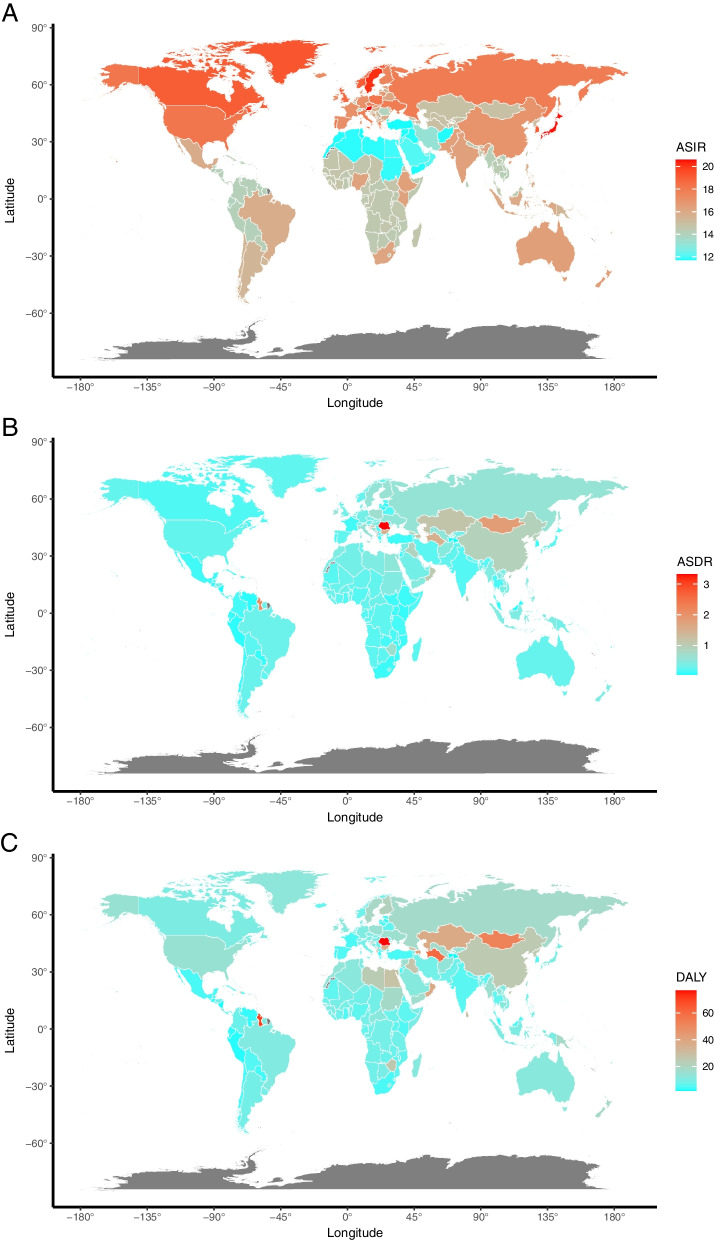
Global disease burden of myocarditis for both sexes in 204 countries and territories. Note: **A** ASIR of myocarditis in 2019; **B** ASDR of myocarditis in 2019; **C** age-standardized DALY rate of myocarditis in 2019

**Fig. 5:**
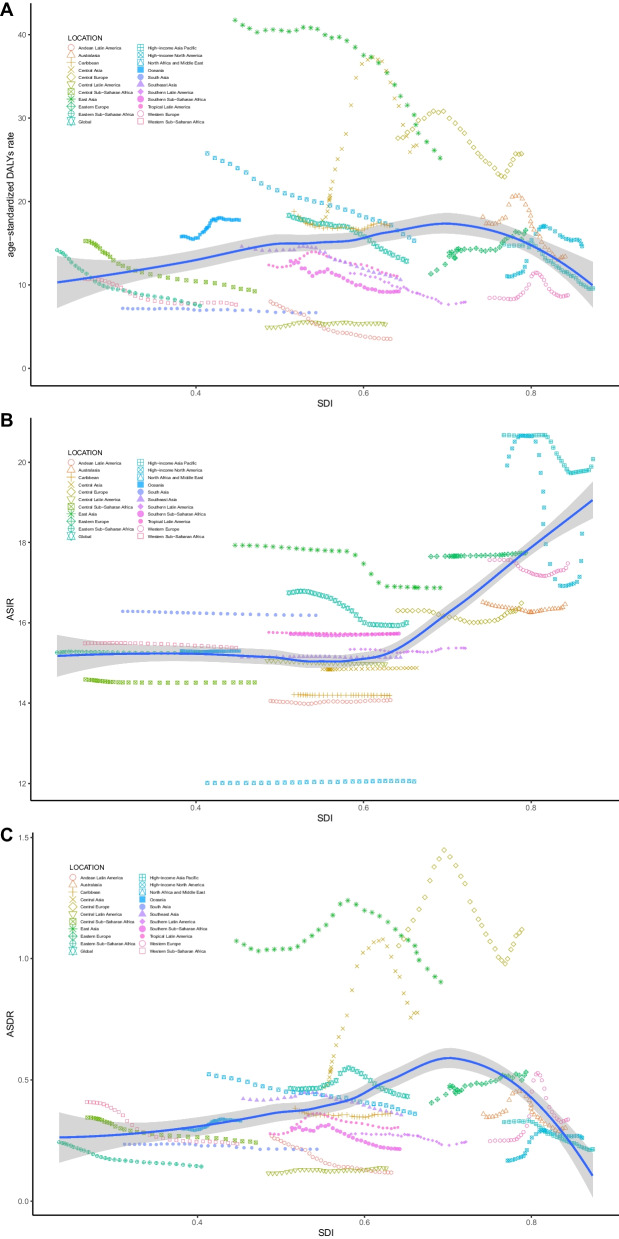
Age-standardized rates of myocarditis among regions based on SDI in 2019. Note: **A** age-standardized DALY rate; **B** ASIR; **C** ASDR

**Fig. 6:**
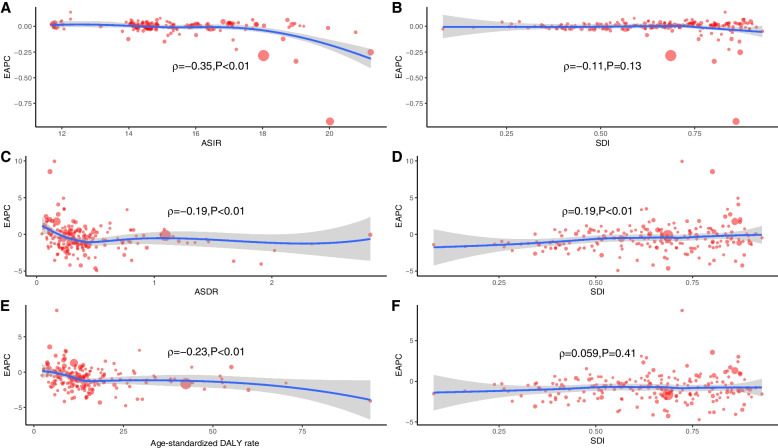
Correlation between EAPC and myocarditis ASR [incidence (**a**), death (**c**), and DALY (**e**)] in 1990 and SDI [incidence (**b**), death(**d**), and DALY (**f**)] in 2019

Globally, the number of the incident cases of myocarditis increased from 780,410 (95%UI, 620,640–951,210) in 1990 to 1265,770 (95%UI, 1021,670–1531,510) in 2019, an increase of 62.19%. In contrast, the ASIR during the past 30 years had a gradually decreasing trend with 16.74/100,000 (95%UI, 13.46–20.34) in 1990 to 16/100,000 (95%UI, 13.01–19.28) in 2019, and the EAPC was -0.23 (from -0.26 to -0.21) (Table 1). As is easily seen from the diagram, the ASIR showed a sharp decrease from 2000 to 2010 compared with the overall tendency (Fig. 1). The factors that might impact ASIR were the age and sex of subjects. The incidence rate and death rate were increasing by the age from 1990 to 2019 (Fig. 2A and B). In 1990, the DALY was stable with age between the age of 5 and 79 (Fig. 2C). Figure 3 gave us a clear impression of how incidence rate, death rate and DALY rate and age were related. Moreover, the gender gap was easily visible in that the incidence rate in females was less compared with that in males (Fig. 3). From data of the 21 regions, the ASIR increased in four regions—Central Asia, Eastern Europe, North Africa, and Middle East—and decreased in eleven regions such as Australasia, Central Latin America, and Central Sub-Saharan Africa; in the other six regions, the ASIR remained stable (Table 1). Globally, North America and most parts of Asia had a higher ASIR, while North Africa had a lower ASIR (Fig. 4). The high-income Asia Pacific had the highest ASIR with an average of 20.21 per 100,000 persons from 1990 to 2019, while North Africa and Middle East (12.03 per 100,000 persons) had the lowest ASIR (Fig. 5). In addition, the study found a weak correlation between EAPC and ASIR ($\rho$=-0.35, *P*<0.01) and a non-significant correlation between EAPC and SDI ($\rho$=-0.11, *P*=0.13) (Fig. 6).

Similarly, the number of total myocarditis deaths increased by 65.40% from 19,618 (95%UI, 15,686–26,772) in 1990 to 32,449 (95%UI, 23,164–37,087) in 2019. Over the three decades, the ASDR was stable with 0.46/100,000 (95%UI, 0.38–0.6) in 1990 to 0.43/100,000 (95%UI, 0.31–0.5) in 2019 (EAPC=-0.99, 95%CI, -0.38 to 0.2) (Table 2). The ASDR in most regions declined, except for six regions (Central Asia, Central Latin America, Eastern Europe, High-income North America, Oceania, and Western Europe) that exhibited rising ASDR and two regions with an ASDR considered to be stable (East Asia and Tropical Latin America) (Table 2). Moreover, the study revealed a very weak correlation between EAPC and ASDR ($\rho$=-0.19, *P*<0.01) and a very weak correlation between EAPC and SDI ($\rho$=0.19, *P*<0.01).

The DALY rate declined by 0.42% from 981,400 (95%UI, 706,950–1578,960) in 1990 to 977,240 (95%UI, 803,760–1126,800) in 2019. Furthermore, the age-standardized DALY rate also showed a decreasing trend from 18.29/100,000 (95%UI, 13.81–27.58) in 1990 to 12.81/100,000 (95%UI, 10.53–14.72) in 2019 (Table 3). Detection of differences of DALY rate in different genders indicated that there were more male subjects than female subjects in most of the age groups, except for those aged 90-94 years and 95+ years. People over 70 years tended to have a lower DALY rate. Interestingly, subjects aged 5–9 years had relatively higher DALY rate (Fig. 2C). Moreover, the study showed a weak correlation between EAPC and DALY ($\rho$=-0.23, *P*<0.01). During the past 30 years, the age-standardized DALY rate was approximately 12, and the EAPC for most of these countries were less than zero level (Fig. 6).

### Myocarditis burden by SDI

Myocarditis burden varies considerably according to five SDI regions in levels and trends from 1990 to 2019. Over the past 30 years, the low SDI region had the highest increasing rate and the average velocity of increase reached 118.17% with ASIR increasing from 15.35 per 100,000 persons (95%UI, 15.07–22.8) in 1990 to 15.29 per 100,000 persons (95%UI, 12.38–18.59) in 2019. In contrast, the high SDI region had the lowest rate of increase among the five different SDI regions. During the 1990 to 2019 study period, the incidences of myocarditis in the high SDI region increased by 42.19% from 172,590 (95%UI, 138,520–210,560) to 245,400 (95%UI, 200,090–298,320), with the ASIR rising form 18.72 per 100,000 persons (95%UI, 15.07–22.8) in 1990 to 17.75 per 100,000 persons (95%UI, 14.71–21.22) in 1990. The most cases were in middle SDI region, while the highest ASIR were in high SDI region. In all five SDI regions, the ASIR showed a decreasing trend from 1990 to 2019 (Table 1).

On the contrary, the high SDI region exhibited the highest increasing rate of death at up to 83.37% during the study period, with 2,321 (95%UI, 18,65–29,31) deaths in 1990 and 4,256 (95%UI, 31,31–49,18) deaths in 2019, with the ASDR rising from 0.26 per 100,000 persons (95%UI, 0.21–0.33) in 1990 to 0.27 per 100,000 persons (95%UI, 0.21–0.31) in 2019. The most cases and the highest ASDRs were both in the middle SDI region. Except for the low-middle SDI region and the low SDI region, the ASDRs in the three other regions were considered to be stable with time. In the low-middle SDI region, the ASDR increased with an EAPC of 0.48 (95%UI, 0.24–0.72), and the annual deaths grew from 2,849 (95%UI, 16.88–39.34) in 1990 to 5,056 (95%UI, 32.14–60.17) in 2019, with the ASDR rising from 0.39 per 100,000 persons (95%UI, 0.28–0.47) in 1990 to 0.41 per 100,000 persons (95%UI, 0.25–0.50) in 2019. In contrast, in the low SDI region, the ASDR decreased with an EAPC of -0.97 (95%UI, from -0.15 to -0.89), and the annual deaths grew from 1,139 (95%UI, 5.67–19.14) in 1990 to 1,663 (95%UI, 11.81–24.62) in 2019 (Table 2), with the ASDR rising from 0.31 per 100,000 persons (95%UI, 0.21–0.44) in 1990 to 0.24 per 100,000 persons (95%UI, 0.15–0.37) in 2019.

The Middle SDI region had the highest value of both DALY and age-standardized DALY rate in 1990 and 2019. During this 30-year study period, there was a decrease in the number of DALY in the middle SDI region; in 1990, there were 425,940 (95%UI, 304,210–742,730) DALY and the age-standardized DALY rate was 25.58/100,000 (95%UI, 19.32–41.28), whereas, in 2019, there were 346,190 (95%UI, 269,340–425,310) DALY and the age-standardized DALY rate was 15.81/100,000 (95%UI, 12.12–19.05). In addition, the low SDI region had the lowest DALY with 73,760 (95%UI, 26,970–149,820) in 1990 and 90,600 (95%UI, 64,780–128,070) in 2019. Among the different SDI regions, the age-standardized DALY rate was considered to be stable in the high SDI region with an EAPC of -0.05 (95%CI, -0.31–0.22) while in other regions, there was a decreasing trend (Table 3).

## Discussion

This study provides an outlook on the incidence, mortality, and DALY of myocarditis from 1990 to 2019, with the hope of informing the strategy for improving the treatment of myocarditis. Results from the study demonstrate the global burden of myocarditis, according to age, sex, and SDI region. The study found that the incidence rate increased by age, the response of the heart to injury is influenced by the sex hormones and high SDI regions with high income tended to have higher values of ASIR. Overall, these results provided reliable and comparatively comprehensive estimates that can optimize the diagnosis, prognosis, and treatment of myocarditis, which should help reduce the evolving global burden of this disease.

The study suggested that the incidence rate increased by age, which also be indicated that the rate of increasing grew faster in people aged 50+ years. Notably, the elderly population tended to have higher values of incidence rate, death rate, and DALY rate, which serves as a reminder to pay attention to the high-risk populations and offer them suitable treatments. Although the total number of incident cases of increased from 1990 to 2019, the ASIR was in a downward trend, which partly reflects the improvement in the treatment of myocarditis [4]. Globally, the death rate was quite low in subjects <75 years, which can be attributed to the baseline condition of each age group since their physical states were not the same. Furthermore, it can be inferred from the study that in middle-SDI regions, the ASDR showed a greater difference between females and males. In the past 30 years, the ASDR exhibited an initial upward trend and then a decreasing trend, and the dividing point was the year 2005. Improved measures for the timely diagnosis and treatment of myocarditis may account for this phenomenon [19]. The DALY rate showed a downward trend, partly because of the improvement of the treatment of myocarditis and the rising level of medical interventions. The DALY rate was higher in the <5 years age group compared with many other age groups (5–65 years), although the disease did not have a higher incidence rate in this age group. Consequently, sufficient attention and medical resources should be allocated more rationally compared with the former proportion for these individuals, especially infants since 16% to 20% of cases of sudden infant death syndrome has something to do with myocarditis [20]. The adoption of such timely measurements will not only improve prognosis but will also lighten the burden for the whole family.

The response of heart to injury is influenced by the sex hormones, which could account for the higher ratio of incidence in male subjects since men were more likely to develop myocarditis [21, 22]. A related study found that myocarditis was more common in men than in women [22]. The prevalence rate of women to men in myocarditis was between 1:1.5 and 1:1.7 [21]. In one study, there were 3198 myocarditis patients and 77% of them were men [22]. The experimental data proves that the sex hormones according to the chromosomal differences are the mean factors that have an influence on the disease [22].

High SDI regions with high income tended to have higher values of ASIR, such as high-income Asia Pacific and North America. Interestingly, the ASIR in many regions (high-income Asia Pacific, high-income North America, Central Europe, Western Europe, and Australasia) had an ascent after an initial decline trend. This phenomenon may be caused by neglection of a diagnosis condition of myocarditis for the low-income districts. The high SDI region may have advanced instruments and equipment to diagnose and treat this kind of disease in early screening of diseases.

Additionally, left ventricular dysfunction indicates a poor recovery in most cases of acute myocarditis [23]. According to a previous study, the primary causes of acute myocarditis were sudden death and chronic dilated cardiomyopathy, especially in Japan and other parts of Asia. In 2009, the combination of acute myocarditis and severe heart failure was linked to a pandemic of influenza (H1N1) [24]. By analyzing hospital dismissal data, the proportion of the heart failure caused by the myocarditis was around 0.5% to 4.0% and varied in people of different age groups and regions [24]. The most common cause of inflammatory cardiomyopathies in developed countries is lymphocytic myocarditis, frequently caused by a viral pathogenesis [25]. However, different causes have been reported such as coxsackieviruses and adenoviruses [26, 27], coronavirus disease-19 related myocarditis [28], diphtheria [29], and typhoid fever [30].

Limitations to the current study were inevitably unavoidable. All the information above was based on the 2019 GBD database, and the accuracy therefore depends on the quality of the data. The statistical errors in data seem unavoidable in criterion of included cases due to different diagnostic conditions. A deficiency of subgroups for this disease prevented us from further researching the discrepancies in disease and providing certain propositions. Moreover, accurate diagnosis is very challenging as myocarditis can be regarded as an inflammation of the myocardium with different clinical symptoms like fatigue, physical decline, chest pain, and palpitations. Timely and accurate diagnosis influences the treatment and prognosis of myocarditis. There are currently marked challenges with the methods used to detect the disease, among which the golden standard for diagnosis is cardiac magnetic resonance imaging. In 2022, Sharifrazi and his research fellows proposed the concept of computer-aided diagnosis to try and address the inaccuracy in the diagnosis of myocarditis [4]. Moreover, little is known about the clinical progression of the disease and further studies are required in this area [31, 32]. American Heart Association stated that timely diagnosis and treatment are crucial to the prognosis of patients with fulminant myocarditis in 2020. Enough attention should be paid in high ASDR regions to get more money for the early diagnosis, precaution and keeping away from risk factors for better prognosis.

## Conclusion

With the improvement of living standards, people shift their emphasis from fundamental demands for pursuing the quality of normal life to guarantee the ultimate goal of living a healthier life that is transformed through disease intervention or prevention. This study provides the latest data from GBD 2019 results, with the main characteristic of comprehensive and realistic information aimed at helping policymakers redistribute the limited human and medical resources to optimize the benefits to the whole world. From 1990 to 2019, the ASIR and DALY decreased, while the ASDR was stable. Elderly people had higher incidence rate and mortality rate of myocarditis. The incidence in male subjects was higher compared with that in female subjects, while the mortality rate showed the opposite trend. The high-middle SDI region and middle SDI region had more deaths compared with the other SDI regions and the trend considered to be comparatively stable. The reason accounted for the situation is that the incident cases are larger than other regions so it can be an enormous burden on the medical system. The patients in these regions may need standardized treatment the ignorance in timely treatment directly contribute to the increasing of the ASDR rate. It is hoped that a decreasing ASDR can be seen in the following years in high-middle and middle SDI regions. More investment and support should be targeted to regions with a high burden of myocarditis, such as East Asia and Central Europe. Further research to investigate the causes of myocarditis will be invaluable to confirm and validate the estimated disease burden.

## Data Availability

The datasets generated during and/or analyzed during the current study are available from the Global Health Data Exchange query tool (http://ghdx.healthdata.org/gbd-results-tool).
